# Sustained efficacy of botulinum toxin in primary palmar hyperhidrosis: a systematic review of duration, quality of life, and satisfaction

**DOI:** 10.3389/fmed.2026.1795512

**Published:** 2026-03-25

**Authors:** Chinonye Eze, Susana Munoz-Lara, Tamunomiete Whyte, Mohammad Jafferany

**Affiliations:** 1 College of Medicine, Central Michigan University, Mt. Pleasant, MI, United States; 2 University of Alabama at Birmingham, Marnix E. Heersink School of Medicine, Birmingham, Alabama, AL, United States; 3 St. George's University School of Medicine, St. George's, Grenada; 4 Department of Psychiatry & Behavioral Sciences, Central Michigan University College of Medicine, Saginaw, MI, United States

**Keywords:** BoNT-A, BoNT-B, botulinum toxin, duration of efficacy, patient improvement, patient satisfaction, primary palmar hyperhidrosis, quality of life

## Abstract

**Background:**

Primary palmar hyperhidrosis (PPH) is a debilitating condition characterized by excessive sweating of the palms, significantly impairing patients’ quality of life (QoL). Botulinum toxin (BoNT) injections are a widely used treatment, but data on their sustained efficacy, impact on QoL, and patient satisfaction remain limited.

**Objective:**

This systematic review aimed to systematically evaluate the duration of BoNT efficacy, its effects on QoL, and patient-reported satisfaction and improvement in PPH.

**Methods:**

This systematic review followed PRISMA guidelines. PubMed, Cochrane Library, and Scopus databases were searched from 2005 to 2024. Studies included randomized controlled trials, observational studies, and clinical trials involving adults with PPH treated with BoNT injections. Outcomes included duration of efficacy, QoL measures (e.g., DLQI), and patient satisfaction.

**Results:**

Nineteen studies met inclusion criteria. The duration of BoNT efficacy ranged from 3 to 12 months, with BoNT-A demonstrating a mean efficacy of 4.9–9.5 months, while BoNT-B showed a shorter duration of 3.8–4.6 months. Higher doses and repeated injections were associated with prolonged symptom control. Disease severity influenced efficacy, with moderate cases experiencing longer relief compared to severe cases. QoL improvements were substantial, with Dermatology Life Quality Index (DLQI) scores showing significant reductions post-treatment. Patient satisfaction rates ranged from 65 to 100%, though this effect diminished over time.

**Conclusion:**

BoNT is an effective and well-tolerated treatment for PPH, offering substantial symptom relief, improved QoL, and high patient satisfaction. Future research should focus on standardized protocols and long-term follow-up to optimize treatment strategies.

## Introduction

Primary palmar hyperhidrosis (PPH) is a chronic condition affecting up to 3% of the population, characterized by excessive sweating of the palms (1). Hyperhidrosis often leads to a significant impact on relationships, mental health, self-esteem, and avoiding social interaction (2). Problems reported with primary palmar hyperhidrosis include writing and fear of holding hands with others (3). Patients with primary hyperhidrosis also reported greater disabilities in work/school, social functioning, and emotional health with up to 48% of patients reporting poor or very poor quality of life (QoL) (2). The stress from social interactions may exacerbate sweat production and in turn perpetuate the cycle of stress and HH, impacting the QoL of patients with HH (2).

Various treatments for PPH exist, including topical antiperspirants, iontophoresis, and surgical interventions (4). However, botulinum toxin (BoNT) injections have emerged as a valuable treatment option for primary focal hyperhidrosis due to their efficacy and favorable safety profile (5). Despite its widespread use, there is considerable variability in treatment protocols, relapse rates, and patient-centered outcomes. This systematic review aims to synthesize existing evidence on the sustained efficacy of BoNT, its impact on QoL, and patient-reported satisfaction and improvement to provide a comprehensive understanding of its role in PPH management.

## Methods

This systematic review was not prospectively registered in a protocol database such as PROSPERO or INPLASY. While registration is recommended to enhance transparency and reduce reporting bias, we have adhered strictly to the PRISMA 2020 guidelines throughout the conduct and reporting of this review to maintain methodological rigor (6).

### PICOS criteria

The Population consisted of individuals diagnosed with primary palmar hyperhidrosis (PPH). The Intervention was intradermal injection of botulinum toxin, including type A (BoNT-A) or type B (BoNT-B). Comparators included placebo, no treatment, or alternative interventions (e.g., iontophoresis). Outcomes of interest were the primary outcome of duration of efficacy (time to relapse, sustained symptom control), and secondary outcomes including quality of life measures (e.g., Dermatology Life Quality Index [DLQI], Patient-Reported Hyperhidrosis Quality of Life [P-HQOL]) and patient satisfaction scores. Eligible Study designs were randomized controlled trials (RCTs), controlled clinical trials, and observational studies with longitudinal data published in English between 2005 and 2024.

### Inclusion and exclusion criteria

Studies were included if they reported on duration of efficacy, quality of life, or patient satisfaction in PPH patients treated with BoNT. Exclusion criteria comprised: case reports, case series, expert opinions, and reviews without primary data; studies on secondary, generalized, or non-palmar hyperhidrosis; studies lacking relevant efficacy, quality-of-life, or satisfaction outcomes; conference abstracts or unpublished data without peer-reviewed validation; and studies utilizing non-needle BoNT delivery methods.

### Search strategy and study selection

A comprehensive search of PubMed, Cochrane Library, and Scopus databases was performed from January 2005 to November 26, 2024. The search was limited to the past two decades to capture studies reflecting modern botulinum toxin formulations, standardized dosing protocols, and contemporary outcome measures (e.g., DLQI, HDSS), which were not consistently reported in earlier literature. This timeframe ensures clinical relevance and applicability to current practice. Keywords and Medical Subject Headings (MeSH) terms included “botulinum toxin,” “palmar hyperhidrosis,” “sweaty palms,” “OnabotulinumtoxinA,” “quality of life,” “patient satisfaction,” and related terms (see Appendix A for the full search strategy). After removal of duplicates, titles and abstracts were screened independently and blindly by three reviewers using Rayyan software (7). Full-text articles of potentially eligible studies were also independently screened by three reviewers and assessed against the inclusion and exclusion criteria. Discrepancies were resolved through discussion and consensus.

### Data extraction and analysis

Data were extracted independently by three reviewers using a standardized form. Extracted information included study characteristics (author, year, design), participant demographics, hyperhidrosis severity, BoNT type and dosage, duration of efficacy, QoL outcomes, satisfaction rates, and adverse events. The risk of bias in RCTs was assessed independently by two reviewers using the Cochrane Risk of Bias Tool and ROBINS-I tool for non-randomized studies of interventions (8, 9). Disagreements were resolved through discussion until a consensus was reached. Summary assessments were generated for each domain, and overall study quality was categorized as low, moderate, or high risk of bias. Risk of bias assessments were used to contextualize findings in the discussion. A summary of risk of bias assessments for all included studies is presented in Tables 1, 2.

**Table 1:** Risk of bias assessment using ROBINS-I tool.

Study (year)	Design	Confounding	Selection	Classification	Deviations	Missing data	Outcome measurement	Selective reporting	Overall
Eilertsen et al. (2024) (31)	Retrospective Study	Serious—Single-arm pre–post design without comparator	Moderate—Single-centre retrospective cohort	Low—Interventions clearly defined and documented.	Moderate—Variation in toxin type, dosing, and anesthesia approach across patients in routine practice	Low—Complete baseline and 2-week DLQI data for all participants	Serious—Subjective self-reported outcomes assessed	Moderate—No pre-registered protocol was reported; however, all stated outcomes appear to have been reported.	Serious
Watanabe et al. (2023) (18)	Prospective Comparative Study	Moderate—Fixed right–left allocation without randomization may introduce side-related bias.	Low—All enrolled patients received both interventions	Low—Interventions clearly defined and documented.	Low—Both delivery methods administered per protocol with standardized dosing	Low—Complete follow-up reported	Serious—Subjective self-reported outcomes assessed	Moderate—No pre-registered protocol was reported; however, all stated outcomes appear to have been reported.	Moderate
Farrell et al. (2021) (26)	Retrospective Study	Serious—No control group; no adjustment for confounders in retrospective single-arm design.	Moderate—Retrospective single-center cohort; unclear if all eligible patients were included.	Low—Interventions clearly defined and documented.	Moderate—No standardized protocol across injectors.	Low—“All patients who received botulinum toxin injections were followed up.”	Serious—Self-reported outcomes collected retrospectively, introducing recall and measurement bias.	Moderate—No pre-registered protocol was reported; however, all stated outcomes appear to have been reported.	Serious
Yang et al. (2018) (17)	Prospective Comparative Study	Serious—Patients self-selected treatment; no statistical adjustment for confounders performed.	Serious—Group allocation based on patient choice rather than randomization.	Low—Interventions clearly defined and documented.	Low—No evidence of deviations from intended interventions	Low—“All patients were followed for 1 year, and completed questionnaires.”	Serious—Subjective self-reported outcomes assessed	Moderate—No pre-registered protocol was reported; however, all stated outcomes appear to have been reported.	Serious
Shayesteh et al. (2016) (3)	Prospective Study	Serious—Single-arm intervention study without control group	Moderate—Consecutive clinic-based sample; tertiary referral population may limit representativeness	Low—Interventions clearly defined and documented.	Moderate—Variation in dosing across patients	Low—Primary hyperhidrosis outcomes were available for all participants; missing data pertained only to secondary quality-of-life measures	Serious—Subjective self-reported outcomes assessed	Moderate—No pre-registered protocol was reported; however, all stated outcomes appear to have been reported.	Serious
D’Epiro et al. (2014) (20)	Prospective Study	Serious—Single-arm design without comparator group	Low—“Consecutive” eligible patients were included according to predefined criteria	Low—Interventions clearly defined and documented.	Moderate—Retreatment performed upon symptom recurrence and open-label design may influence outcome assessment	Low—No loss to follow-up or missing outcome data reported across 12-month assessment.	Serious—Subjective self-reported outcomes assessed	Moderate—No pre-registered protocol was reported; however, all stated outcomes appear to have been reported.	Serious
Lecouflet et al. (2014) (11)	Retrospective Study	Serious—Single-arm design without comparator	Low—All eligible clinic patients included over defined period	Low—Interventions clearly defined and documented.	Low—No evidence of systematic protocol deviation	Moderate—Long follow-up window; No explicit attrition reporting.	Serious—Subjective self-reported outcomes assessed	Moderate—No pre-registered protocol was reported; however, all stated outcomes appear to have been reported.	Serious
Rajagopal and Mallya (2014) (16)	Prospective Comparative Study	Serious—Predicatable Sequence of allocation	Serious—Allocation could be influenced	Low—Interventions clearly defined and documented.	Moderate—Open-label design with crossover permitted at 4 weeks	Low—No loss to follow-up or missing HDSS data reported at 4-week assessment.	Serious—Subjective self-reported outcomes assessed	Moderate—No pre-registered protocol was reported; however, all stated outcomes appear to have been reported.	Serious
Campanati et al. (2013) (22)	Prospective Controlled Study	Moderate—Intra-individual design reduces between-subject confounding; however, allocation was non-randomized.	Low—Participants prospectively enrolled according to predefined objective and clinical criteria	Low—Interventions clearly defined and documented.	Low—Both treatments administered simultaneously per protocol with no reported deviations	Low—Follow-up completed at predefined time points with no reported loss to follow-up.	Serious—Subjective self-reported outcomes assessed	Moderate—No pre-registered protocol was reported; however, all stated outcomes appear to have been reported.	Serious
El Kahky et al. (2013) (29)	Prospective, Comparative Study	Moderate—Fixed right–left allocation without randomization may introduce side-related bias.	Moderate—Small pilot sample without explicit consecutive enrollment; representativeness of the source population unclear.	Low—Interventions clearly defined and documented.	Low—Both treatments administered simultaneously per protocol with no reported deviations	Low—Follow-up completed at predefined time points with no reported loss to follow-up.	Serious—Subjective self-reported outcomes assessed	Moderate—No pre-registered protocol was reported; however, all stated outcomes appear to have been reported.	Serious
Ito et al. (2011) (14)	Prospective Study	Serious—Single-arm design without comparator	Moderate—Participants recruited from a single centre without explicit statement of consecutive inclusion	Low—Interventions clearly defined and documented.	Low—Treatment delivered according to standardized protocol with no reported deviations	Low—Follow-up completed at predefined time points with no reported loss to follow-up.	Low—Sweat production assessed objectively using standardized measurements	Moderate—No pre-registered protocol was reported; however, all stated outcomes appear to have been reported.	Serious
Campanati et al. (2011) (25)	Prospective Study	Serious—Single-arm design without comparator	Moderate—Participants were enrolled from routine clinical practice during a defined period, but consecutive inclusion not reported	Low—Interventions clearly defined and documented.	Low—Treatment delivered according to standardized protocol with no reported deviations	Low—Follow-up completed at predefined time points with no reported loss to follow-up.	Moderate—Outcomes included subjective HDSS/DLQI and clinician-scored Minor test performed by an external clinician	Moderate—No pre-registered protocol was reported; however, all stated outcomes appear to have been reported.	Serious
Martí et al. (2010) (10)	Prospective Study	Serious—Single-arm design without comparator	Moderate—Participants were enrolled prospectively during a defined period, but consecutive inclusion not reported and inclusion required questionnaire completion	Low—Interventions clearly defined and documented.	Low—Treatment delivered according to standardized protocol with no reported deviations	Low—No loss to follow-up or missing outcome data reported	Serious—Subjective self-reported outcomes assessed.	Moderate—No pre-registered protocol was reported; however, all stated outcomes appear to have been reported.	Serious
Aghaei et al. (2007) (15)	Non-randomized Interventional Study	Serious—Single-arm design without comparator	Moderate—Patients were recruited from dermatology clinics using predefined criteria, but, consecutive inclusion and screening processes were not described.	Moderate—Intervention dosing differed between dominant and nondominant hands.	Low—Treatment delivered according to standardized protocol with no reported deviations	Low—No loss to follow-up or missing outcome data reported	Serious—Subjective self-reported outcomes assessed.	Moderate—No pre-registered protocol was reported; however, all stated outcomes appear to have been reported.	Serious
Pérez-Bernal et al. (2005) (21)	Prospective Study	Serious—Single-arm design without comparator; reinjection timing was patient-driven and natural disease course was not controlled	Serious—Of 89 treated patients, only 69 were included in analysis without explanation of exclusions	Low—Interventions clearly defined and documented.	Moderate—Reinjection timing was based on patient request rather than standardized criteria	Serious—20 patients were not included in the analysis and completeness of follow-up across timepoints was not reported	Serious—Subjective self-reported outcomes assessed	Moderate—No pre-registered protocol was reported; however, all stated outcomes appear to have been reported.	Serious
Basciani et al. (2014) (13)	Prospective Study	Serious—Single-arm design without comparator	Moderate—Patients were prospectively screened using predefined criteria; representativeness of the enrolled sample not fully described	Low—Interventions clearly defined and documented.	Low—Treatment delivered according to standardized protocol with no reported deviations	Low—No loss to follow-up or missing outcome data reported across scheduled assessments	Serious—Subjective self-reported outcomes assessed.	Moderate—No pre-registered protocol was reported; however, all stated outcomes appear to have been reported.	Serious
Rosell et al. (2013) (19)	Prospective Study	Serious—Single-arm design without comparator	Low—Consecutive patients included according to predefined criteria during a defined recruitment period.	Low—Interventions clearly defined and documented.	Low—Treatment delivered according to standardized protocol with no reported deviations	Low—No loss to follow-up or missing outcome data reported	Serious—Subjective self-reported outcomes assessed.	Moderate—No pre-registered protocol was reported; however, all stated outcomes appear to have been reported.	Serious

**Table 2:** Risk of bias assessment using Cochrane RoB 2 tool.

Study (year)	Design	Randomization	Deviations from intended intervention	Missing data	Outcome measurement	Selection of reported result	Overall
Alhetheli (2021) (23)	Parallel Group RCT	Low—Random allocation using opaque envelopes; baseline characteristics comparable between groups	Some Concern—Open-label design; however, ITT analysis performed.	Low—No missing outcome data reported	Some Concern—Subjective outcomes assessed in open-label setting; assessor blinding not reported	Some Concern—No trial registration or pre-specified protocol reported.	Some Concerns
Baumann et al. (2005) (12)	RCT	Low—Randomization was performed by an independent nurse and baseline characteristics were comparable between groups.	Low—Participants and investigators were blinded and deviations from assigned intervention were minimal prior to the primary endpoint.	Low—Missing occurred after day 30 (primary endpoint)	Low—Outcomes were assessed in a double-blind manner using standardized questionnaires and blinded physician photo assessment.	Some Concern—No evidence of selective reporting, but no registry either	Some Concerns

### Data synthesis

Due to anticipated heterogeneity in study designs, interventions (BoNT type/formulations, doses, injection techniques), and outcome measures (variable definitions of ‘duration,’ different QoL instruments, non-standardized satisfaction scales), a meta-analysis was not feasible. Heterogeneity was assessed qualitatively by examining differences in study populations, interventions, comparators, outcomes, follow-up duration, study designs, and risk of bias. Where appropriate, simple pooled means were calculated for descriptive purposes, though these should be interpreted cautiously given the underlying heterogeneity. A narrative synthesis was performed, organizing findings by the primary themes of duration of efficacy and quality of life and patient satisfaction. Subgroup considerations included toxin type (BoNT-A vs. BoNT-B vs. BoNT-A/B), dosage, and baseline disease severity.

## Results

### Study selection

**Figure 1:**
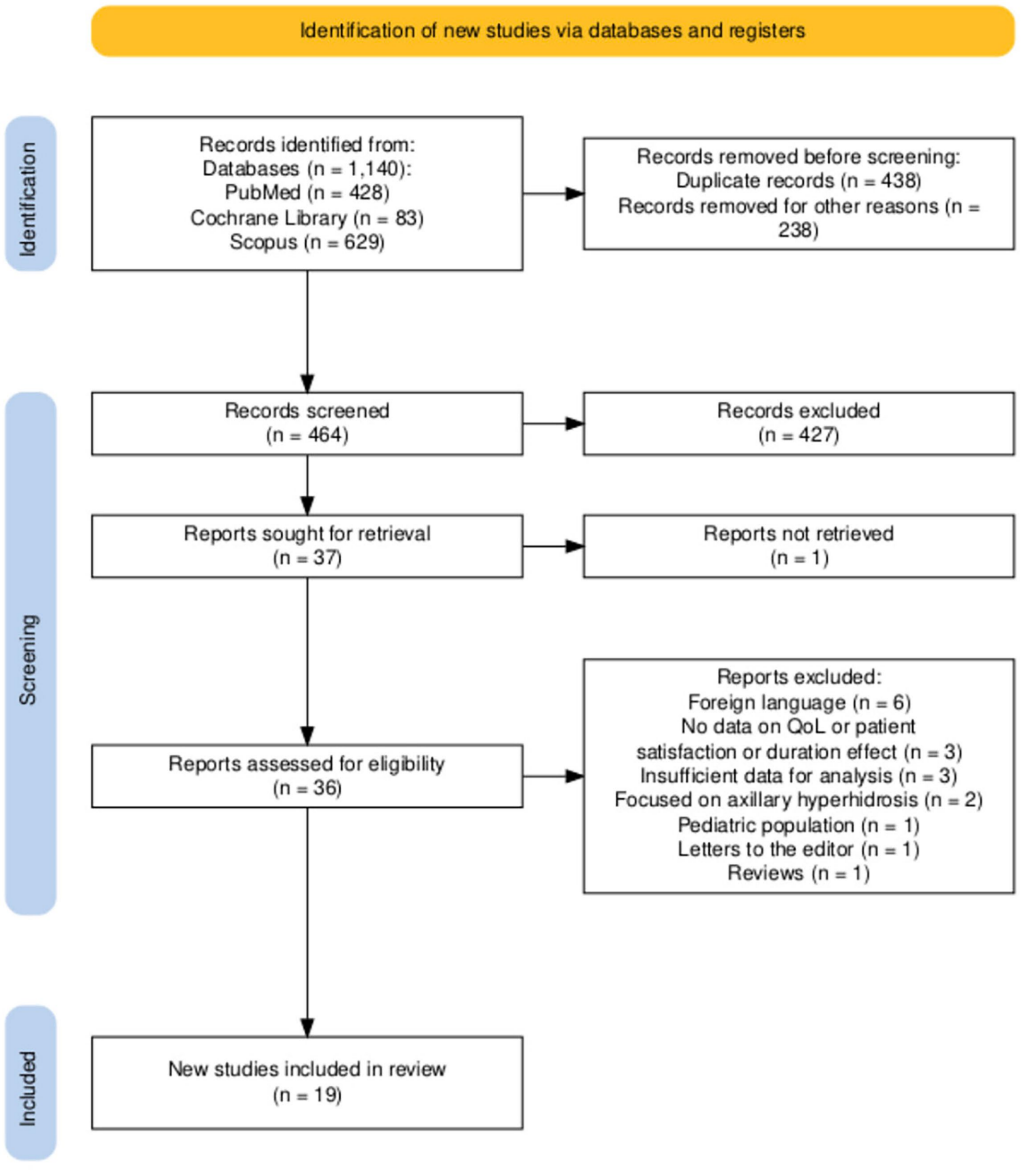
PRISMA flow diagram.

The study selection process is detailed in the PRISMA flow diagram (Figure 1). A total of 1,140 records were identified through database searching (PubMed: *n* = 428; Cochrane Library: *n* = 83; Scopus: *n* = 629). Prior to screening, 676 records were removed, including duplicate records (*n* = 438) and records excluded due to publication date prior to 2005 (*n* = 238), as pre-specified in our search parameters to ensure clinical relevance. The remaining 464 records underwent title and abstract screening, during which 427 records were excluded for reasons including: not related to primary palmar hyperhidrosis, not involving botulinum toxin intervention, and wrong publication type (e.g., reviews, case reports, editorials). Of the 37 reports sought for retrieval, one could not be obtained. The remaining 36 reports underwent full-text eligibility assessment, with 17 reports excluded for the following reasons: foreign language (*n* = 6), no data on quality of life, patient satisfaction, or duration of efficacy (*n* = 3), insufficient data for analysis (*n* = 3), focused on axillary hyperhidrosis (*n* = 2), pediatric population (*n* = 1), letters to the editor (*n* = 1), and reviews (*n* = 1). Nineteen studies were included in the final synthesis.

### Duration of efficacy

The duration of BoNT efficacy in treating PPH ranged from 3 to 12 months, with several factors influencing the length of symptom control. As summarized in Table 3, BoNT-A, the most commonly studied formulation (14 studies), demonstrated a mean duration of efficacy ranging from 4.9 to 9.5 months with a weighted mean of approximately 6.2 months across studies (10, 11). Repeated injections BoNT were associated with prolonged efficacy, increasing from a median of 7 months after the first treatment to 9.5 months after the last injection (*p* = 0.0002), highlighting the benefits of retreatment (11). In contrast, BoNT-B showed a shorter duration of efficacy, with mean symptom control lasting 3.8 to 4.6 months (12, 13). The weighted mean for BoNT-B was approximately 4.2 months, notably shorter than BoNT-A.

**Table 3:** Duration of efficacy by study.

Study (year)	Study design	Toxin type	Toxin dose/concentration	Follow-up duration	Sample size	Duration of efficacy (months)	Key findings
Alhetheli (2021) (23)	Parallel Group RCT	BoNT-A	Botox 5 IU/mL	3 months	20	12 weeks	Mean duration: 12 weeks; higher satisfaction in BoNT group.
Farrell et al. (2021) (26)	Retrospective Study	BoNT-A	Onabotulinum toxin A 100 U/12 mL per palm	NR	30	4 (median)	Median duration: 4 months (range: 1–14 months); increased with repeated injections.
Yang et al. (2018) (17)	Prospective Comparative Study	BoNT-A	BTX-A 2 U/0.1 mL/cm^2^	12 months	26	~6 months	Recurrence of symptoms in 61.5% at 3 months and 80.8% at 6 months.
Shayesteh et al. (2016) (3)	Prospective Study	BoNT-A	Botox 120–160 U/palm	NR	39	9.1 ± 4.3 (mean)	Mean duration: 9.1 ± 4.3 months.
D’Epiro et al. (2014) (20)	Prospective study	BoNT-A	Botox 2 MU/2.25 cm^2^	12 months	26	4.27 (mean)	Mean duration: 4.27 months.
Lecouflet et al. (2014) (11)	Retrospective Study	BoNT-A	Dysport 250 U/palm	5 months – 9 years (mean – 3.92 years)	28	7 → 9.5 (repeated)	Duration increased from 7 to 9.5 months with repeated injections (*p* = 0.0002).
Rajagopal and Mallya (2014) (16)	Prospective Comparative Study	BoNT-A	Botulinum toxin A 100 U/palm	6 months	30	4 (mean)	Mean duration: 4 months; 80% improvement in initial cases.
El Kahky et al. (2013) (29)	Prospective, Comparative Study	BoNT-A	Botox 50 U x 1 palm vs. Dysport 125 U x 1 palm	8 months	**8**	4–6 months	Mean duration: 4–6 months for both Botox and Dysport.
Ito et al. (2011) (14)	Prospective Study	BoNT-A	Botox 90 U x 1 palm	7 months	29	6.44 (moderate), 3.33 (severe)	Moderate cases: 6.44 ± 1.03 months; severe cases: 3.33 ± 1.32 months.
Campanati et al. (2011) (25)	Prospective Study	BoNT-A	Botox 2 MU/2.25 cm^2^	15 months	41	<6 months (relapse-free)	Relapse-free duration <6 months; shorter in patients with >20 years of disease.
Martí et al. (2010) (10)	Prospective Study	BoNT-A	Botox 100 MU/palm	12 months	22	4.9 ± 1.9 (mean)	Mean duration: 4.9 ± 1.9 months; median: 5 months (range: 1–9 months).
Aghaei et al. (2007) (15)	RCT	BoNT-A	Dysport Dominant hand: 10 U/cm^2^ Nondominant hand: 5 U/cm^2^	12 months	**14**	5.42 (anhidrosis)	Mean duration of anhidrosis: 5.42 ± 1.34 months; hypohidrosis: 10.43 ± 1.45 months.
Pérez-Bernal et al. (2005) (21)	Prospective Study	BoNT-A	Botox 80–100 U/palm	44 months	69	3–7 months	Maximum improvement lasted up to 3 months; reinjections needed after 7 months.
Rosell et al. (2013) (19)	Prospective Study	BoNT-A/B	Xeomin 213 ± 19 U (Mean)/patient Neurobloc 264 ± 60 U (Mean)/patient	NR	26	5.2 (median)	Median duration: 5.2 months (range: 4.2–6.9 months).
Basciani et al. (2014) (13)	Prospective Study	BoNT-B	Neurobloc 5,000 IU/ palm	6 months	32	4.6 ± 0.8 (mean)	Mean duration: 4.6 ± 0.8 months (range: 3–6 months).
Baumann et al. (2005) (12)	RCT	BoNT-B	Myobloc 5,000 U/palm *Vs* Placebo	3 months (Extended Follow-up till patients returned to baseline)	20	3.8 (mean)	Mean duration: 3.8 months (range: 2.3–4.9 months).

Bolded sample sizes indicate studies with *N* < 20 (limited statistical power).

RCT, Randomized Controlled Trial; NR, Not Reported.

Disease severity also played a significant role in determining the duration of efficacy. Patients with moderate hyperhidrosis (HDSS 3) experienced longer symptom control (6.44 ± 1.03 months) compared to those with severe hyperhidrosis (3.33 ± 1.32 months) (14). Additionally, higher doses of BoNT, such as 500 U of Dysport®, were associated with extended periods of anhidrosis (5.42 ± 1.34 months) (15), while lower doses (e.g., 100 U per palm) yielded shorter durations of approximately 4 months (16). Recurrence of symptoms was observed in 61.5% of patients at 3 months and 80.8% at 6 months post-treatment (17), emphasizing the need for individualized treatment protocols.

### Quality of life, patient reported satisfaction, and improvement

BoNT therapy significantly improved QoL and patient satisfaction across studies, as detailed in Table 4. The Dermatology Life Quality Index (DLQI) scores showed median reductions from 9.69 ± 4.09–20.9 pre-treatment to 1.2–7 post-treatment, indicating substantial improvements in patients’ QoL (18–20).

**Table 4:** Patient-reported satisfaction, improvement, and quality of life.

Study (year)	Study design	Sample size	Toxin type	Toxin dose/concentration	Follow-up duration	Quality of life (pre → post)	Patient satisfaction rate	Improvement rate
Watanabe et al. (2023) (18)	Prospective Comparative Study	**16**	BoNT-A	Botox Vista 100 U/palm	Up to 28 weeks	Mean DLQI: 9.69 ± 4.09 → 1.94 ± 1.29	77.6%	NR
Alhetheli (2021) (23)	Parallel Group RCT	20	BoNT-A	Botox 5 IU/ml	3 months	NR	75%	NR
Rajagopal and Mallya (2014) (16)	Prospective Comparative Study	30	BoNT-A	Botulinum toxin A 100 U/palm	6 months	NR	NR	80% improvement in initial cases; significant improvement in HDSS 4 cases.
D’Epiro et al. (2014) (20)	Prospective Study	26	BoNT-A	Botox 2 MU/2.25 cm^2^	12 months	DLQI: 20.9 → 7	85% satisfaction	46% achieved HDSS 1
El Kahky et al. (2013) (29)	Prospective, Comparative Study	**8**	BoNT-A	Botox 50 U x 1 palm vs. Dysport 125 U x 1 palm	8 months	DLQI improved for both Botox and Dysport (*p* < 0.05)	No significant difference in satisfaction	NR
Campanati et al. (2013) (22)	Prospective Controlled Study	50	BoNT-A	Botox 100–150 MU/ hand	6 months	NR	Higher satisfaction with needle adapter method (mean score: 2.42 vs. 1.82, *p* < 0.0001)	NR
Campanati et al. (2011) (25)	Prospective Study	41	BoNT-A	Botox 2 MU/2.25cm^2^	15 months	DLQI: 15 (for palmar) and 14 (general HH) → 1 (*p* < 0.001).	NR	NR
Aghaei et al. (2007) (15)	RCT	**14**	BoNT-A	Dysport Dominant hand: 10 U/cm^2^ Nondominant hand: 5 U/cm^2^	12 months	NR	Ranged from 30 to 90% (mean: 65%)	NR
Pérez-Bernal et al. (2005) (21)	Prospective Study	69	BoNT-A	Botox 80–100 U/palm	44 months	NR	NR	85.5% of patients maintained >60% improvement at 1 month; declined to 26% at 6 months.
Eilertsen et al. (2024) (31)	Retrospective Study	**13**	BoNT-A/B	BTX-A 400 IU (Median)/patient And BTX-B 200 IU (Median)/patient	2 weeks	Median DLQI: 10.5 reduction (*p* = 0.001)	NR	NR
Rosell et al. (2013) (19)	Prospective Study	26	BoNT-A/B	Xeomin 213 ± 19 U (Mean)/patient Neurobloc 264 ± 60 U (Mean)/ patient	NR	DLQI: 10.3 → 1.2 (*p* < 0.05)	100% of patients satisfied (≥4/5)	NR
Baumann et al. (2005) (12)	RCT	20	BoNT-B	Myobloc 5,000 U/palm *Vs* Placebo	3 months (Extended Follow-up till patients returned to baseline)	Significant improvement in P-HQOL scores (*p* = 0.010)	NR	Significant improvement in P-HI (*p* = 0.002)

RCT, Randomized Controlled Trial; DLQI, Dermatology Life Quality Index; HDSS, Hyperhidrosis Disease Severity Scale; P-HQOL, Patient-Reported Hyperhidrosis Quality of Life; P-HI, Patient-Reported Hyperhidrosis Improvement; NR, Not Reported.

Bolded sample sizes indicate studies with *N* < 20 (limited statistical power).

Some studies reported multiple outcomes; only those relevant to patient-reported measures are included here.

DLQI scores are presented as mean unless otherwise specified. Lower scores indicate better quality of life (scale 0–30).

HDSS is a 4-point scale where lower scores indicate less severe hyperhidrosis.

P-HQOL and P-HI are validated instruments for hyperhidrosis-specific quality of life and improvement.

BoNT provided mean patient satisfaction rates ranging from 65 to 100%, with 100% of patients in one study reporting satisfaction levels of 4 or 5 on a 5-point scale (19). In another study, 85.5% of patients reported “excellent improvement” or “good improvement” at 1 month post-treatment, though this declined to 26% by 6 months (21). Most studies reporting rates above 75% as reported in Table 4. Notably, Campanati et al. (22) demonstrated that injection technique significantly influences patient experience, with higher satisfaction using a needle adapter method compared to standard injection (mean score: 2.42 vs. 1.82, *p* < 0.0001). This finding suggests that optimizing delivery methods may enhance patient acceptance and treatment adherence.

Improvements in the Hyperhidrosis Disease Severity Scale (HDSS) were also noted, with 46% of patients achieving HDSS 1 (mild or no symptoms) after treatment (20). Baumann et al. (12) used validated instruments, demonstrating significant improvement in Patient-Reported Hyperhidrosis Improvement (P-HI) scores (*p* = 0.002), providing robust evidence of patient-perceived benefit. Comparative studies found that BoNT was superior (*p* = 0.007) to alternative treatments such as iontophoresis, with 80% of patients reporting improvement compared to 46.7% in the iontophoresis group (16).

### Risk of bias assessment

Risk of bias was assessed using the Cochrane RoB 2 tool for RCTs and ROBINS-I for non-randomized studies (Tables 1, 2). The two included RCTs (12, 23) were both rated as having “some concerns” overall due to open-label designs and lack of pre-registered protocols, though randomization and blinding were adequate (12, 23). Among the 17 non-randomized studies, overall risk of bias was rated as serious for 16 studies and moderate for one. The predominance of serious ratings reflects consistent limitations across studies: single-arm designs lacking comparator groups, reliance on subjective patient-reported outcomes, and absence of pre-registered protocols. However, classification of interventions was consistently low risk, and missing data were minimal in most studies. Despite these methodological limitations, the consistency of findings across studies, particularly for QoL improvements and satisfaction, may suggest robust treatment effects.

## Discussion

This systematic review highlights the duration of efficacy of BoNT in managing PPH, with significant improvements in symptom control, quality of life (QoL), and patient satisfaction. The duration of efficacy varied based on toxin type, dosage, and disease severity, with BoNT-A generally outperforming BoNT-B in terms of sustained symptom relief (11, 12).

As shown in Table 4, BoNT therapy significantly improved QoL and patient satisfaction across studies. The reduction in DLQI scores from pre-treatment values of 9.69 ± 4.09–20.9 to post-treatment values of 1.2–7 reflects the therapy’s ability to address both the physical and emotional burdens of PPH (18–20). Patient satisfaction rates ranged from 65 to 100%, with Rosell et al. (19) reporting 100% satisfaction among palmar patients treated with BoNT-A. Comparative analyses revealed BoNT’s superiority over alternative treatments like iontophoresis, particularly in severe PPH cases (16). Studies with longer follow-up periods reported a gradual decline in patient satisfaction, likely due to symptom recurrence (21).

### Comparison with previous literature

Our findings align with previous systematic reviews, including that by Galadari et al., which reported that the mean or median duration of effect for abobotulinumtoxinA (aboBoNT-A) in the treatment of palmar hyperhidrosis ranged from 3 to 10 months (24). Galadari et al. also found that patient satisfaction was high and significant improvements to quality of life were observed after aboBoNT-A treatment.

### Optimizing treatment response: dosing strategies and retreatment patterns

The variability in dosing protocols across studies reflects the absence of standardized guidelines, but clinically relevant patterns emerged from the aggregated data. In our review, the observed dose range for Botox (50–160 U per palm) and Dysport (125–250 U per palm) when evaluating duration of efficacy suggests that clinicians have considerable flexibility to individualize treatment based on patient characteristics (See Table 3). Higher doses were often associated with longer duration of efficacy as seen in Aghaei et al. which found dominant hand dosing at 10 U/cm^2^ produced longer anhidrosis compared to non-dominant hand dosing at 5 U/cm^2^ (5.42 vs. 10.43 months for hypohidrosis) (15). However, the relationship between dose and duration is not linear, and appears modified by patient-specific factors that warrant consideration in treatment planning.

The finding that patients with moderate disease (HDSS 3) experienced nearly double the duration of efficacy compared to those with severe disease (HDSS 4) (6.44 vs. 3.33 months), carries important clinical implications (14). This suggests that severe cases may require more frequent retreatment regardless of dose, and clinicians should set realistic expectations accordingly. For patients with mild-to-moderate disease, starting at the lower end of the dose range (Botox 50–100 U per palm) may achieve adequate symptom control while minimizing muscle weakness risk, with dose escalation reserved for those with suboptimal response. For severe disease, higher initial doses (Botox 120–160 U per palm) may be warranted despite the shorter expected interval between treatments.

Regarding retreatment, Lecouflet et al. provided compelling evidence for cumulative benefits, demonstrating that the median duration of efficacy of BoNT increased significantly from 7 months after the first injection to 9.5 months after the last injection (*p* = 0.0002) (11). This cumulative benefit suggests that the full therapeutic potential of BoNT may not be realized until after several treatment cycles. This has important implications for both patient counseling and healthcare resource planning. Patients who discontinue treatment after one or two sessions due to perceived “limited duration” may never achieve the extended intervals possible with continued therapy. Clinicians should educate patients that treatment response may improve over time and that commitment to a series of injections may yield progressively greater convenience and cost-effectiveness.

Disease severity significantly influenced treatment outcomes beyond dosing alone. Campanati et al. noted that relapse-free duration was <6 months and was shorter in patients with >20 years of disease, suggesting that chronicity may impact treatment responsiveness (25). This suggests that longstanding hyperhidrosis may be associated with glandular changes that render it more resistant to treatment, or that patient expectations and symptom perception may differ with chronicity. Clinically, this highlights the importance of early intervention in the disease course to optimize long-term outcomes. For patients with chronic disease, more intensive initial dosing or combination approaches may be necessary.

### Adverse effects and safety profile

In our review, adverse effects associated with BoNT therapy for PPH were generally mild and transient based on the palmar-specific studies included in this review. The most commonly reported side effects in palmar studies included muscle weakness (23–60% of patients) and dry hands (60%) (12, 26). Severe, yet rare, complications such as functional grip impairment were specifically reported in PPH patients (26). Clinicians should assess individual patient factors, particularly occupational demands, as grip weakness may disproportionately impact patients in manual or fine motor skill-dependent professions compared to those in less manually demanding roles. Modified injection techniques, along with appropriate dosing strategies, may help mitigate this risk while maintaining therapeutic efficacy. Patients may benefit from more literature addressing techniques that mitigate outcomes that impede manual functionality.

It is important to note that while the safety profile of BoNT in palmar hyperhidrosis is consistent with the established safety profile in axillary hyperhidrosis (27), direct extrapolation should be cautious due to anatomical and functional differences between palmar and axillary treatment sites. The palmar region presents unique considerations including higher density of nerve endings (affecting pain), greater risk of muscle weakness affecting hand function, and different skin thickness affecting injection technique. Also, in axillary hyperhidrosis, BoNT is widely established as first-line therapy with robust evidence from multiple large RCTs. In contrast, for PPH, the evidence base is more limited, and BoNT should be considered an effective treatment option rather than a universal gold standard. PPH presents unique challenges including the mechanical demands of hand function, higher pain sensitivity in palms, and greater variability in treatment response. These distinctions are critical when counseling patients and when extrapolating treatment guidelines from axillary to palmar indications.

### Comparative cost effectiveness

While no formal cost-effectiveness analyses specific to palmar hyperhidrosis, the axillary hyperhidrosis model by Gibbons et al. offers a useful framework for understanding the economic trade-offs between botulinum toxin and surgical intervention (28). Their analysis revealed that BoNT, despite requiring repeated treatments every 5–6 months at an annual cost of €853, would take over 13 years to reach cost equivalence with endoscopic thoracic sympathectomy (ETS) (28). This also takes into account the additional cost of complications associated with ETS including risks including pneumothorax (0.4–2.3%), compensatory sweating (50–70%), and Horner’s syndrome (1%) (28). This suggests that from an economic standpoint, BoNT remains competitive with surgery, particularly when one considers surgical complications that may require ongoing management. However, this axillary-derived model likely underestimates the cost advantage of BoNT in palmar disease, where functional impairment may carry occupational and quality-of-life costs not captured in axillary analyses. Therefore, clinicians should weigh these preliminary findings against individual patient factors.

### Limitations

Despite the promising findings, several limitations must be acknowledged. First, the heterogeneity in study designs, including variations in toxin type, dosage, and injection techniques, limits the ability to draw definitive conclusions. For example, some studies used BoNT-A (e.g., Dysport®, Botox®), others used BoNT-B (e.g., Myobloc®), some even used both making direct comparisons challenging. Second, small sample sizes in many studies [e.g., (29); *n* = 8] reduce the statistical power and generalizability of the findings. Third, the lack of long-term follow-up data in most studies limits our understanding of the durability of BoNT therapy beyond 6–12 months. Fourth, and importantly, the overall quality of the evidence presents significant limitations. The majority of included studies were non-randomized with serious risk of bias (Tables 1, 2). Only two RCTs were identified, both with some concerns. This methodological landscape means that while the consistency of findings supports BoNT’s efficacy, the magnitude of subjective benefits may be overestimated. Other limitations of this review include variability among study designs, ranging from double-blind, randomized, placebo-controlled studies to observational studies, and inconsistent dosing protocols, which may affect the generalizability of findings. Additionally, patient-reported outcomes, while valuable, introduced subjective biases. Another limitation is the underreporting of adverse effects. While transient muscle weakness and injection-site pain were commonly noted, long-term side effects were seldom evaluated. This gap in data calls for future research focusing on the long-term safety of BoNT treatments in PPH.

### Future directions

Future research should focus on large-scale, randomized controlled trials (RCTs) comparing different BoNT formulations, standardized dosing regimens, and injection techniques. Studies should also explore the impact of repeated injections on long-term efficacy as well as antibody formation, which has been reported in a small subset of patients in axillary hyperhidrosis literature (30). Additionally, the development of novel delivery methods, such as needle-free jet injectors, may improve patient comfort and adherence to treatment (18).

## Conclusion

Botulinum toxin is an effective and well-tolerated treatment for primary palmar hyperhidrosis, offering substantial symptom relief, improved quality of life, and high patient satisfaction. However, the current body of literature is limited by small sample sizes, short follow-up periods, and heterogeneity in study methodologies. Future research should focus on large-scale, randomized controlled trials with standardized protocols to optimize treatment regimens and improve comparability across studies. Long-term follow-up studies are also essential to evaluate the cumulative effects, cost-effectiveness, and potential long-term adverse effects of BoNT therapy. Addressing these gaps will further solidify BoNT’s role in the management of PPH and improve patient-centered outcomes.

## Data Availability

The original contributions presented in the study are included in the article/supplementary material, further inquiries can be directed to the corresponding author.

## Appendix A: Search terms

Database Search Terms on November 26, 2024.

### PubMed search (428 results)

(“Botulinum Toxins, Type A”[Mesh] OR “Iontophoresis”[Mesh] OR “Antiperspirants”[Mesh] OR “botulinum toxin*”[tiab] OR “botulinum A toxin*”[tiab] OR “botulinum neurotoxin*”[tiab] OR Botox[tiab] OR Oculinum[tiab] OR “OnabotulinumtoxinA”[tiab] OR “Onabotulinumtoxin A”[tiab] OR Meditoxin[tiab] OR Vistabel[tiab] OR Neuronox[tiab] OR iontophores*[tiab] OR antiperspirant*[tiab] OR “aluminum chloride”[tiab] OR “topical aluminum chloride”[tiab]) AND (“Hyperhidrosis Palmaris Et Plantaris”[Supplementary Concept] OR “palmar hyperhidros*”[tiab] OR “hyperhidrosis palmar*”[tiab] OR “sweaty palm*”[tiab] OR ((“Hyperhidrosis”[Mesh] OR “Sweating”[Mesh] OR hyperhidros*[tiab] OR sweat*[tiab]) AND (“Hand”[Mesh] OR palm*[tiab] OR hand*[tiab]))).

### Cochrane library search (83 results)

([mh “Botulinum Toxins, Type A”] OR [mh Iontophoresis] OR [mh Antiperspirants] OR (“botulinum” NEXT toxin*):ti,ab OR (“botulinum A” NEXT toxin*):ti,ab OR (“botulinum” NEXT neurotoxin*):ti,ab OR Botox:ti,ab OR Oculinum:ti,ab OR OnabotulinumtoxinA:ti,ab OR “Onabotulinumtoxin A”:ti,ab OR Meditoxin:ti,ab OR Vistabel:ti,ab OR Neuronox:ti,ab OR iontophores*:ti,ab OR antiperspirant*:ti,ab OR (“topical” NEXT “aluminum chloride”):ti,ab OR “aluminium chloride hexahydrate”:ti,ab OR “Drysol”:ti,ab OR “Xerac AC”:ti,ab) AND ((“palmar” NEXT hyperhidros*):ti,ab OR (“hyperhidrosis” NEXT palmar*):ti,ab OR (“sweaty” NEXT palm*):ti,ab OR (([mh Hyperhidrosis] OR [mh Sweating] OR hyperhidros*:ti,ab OR sweat*:ti,ab) AND ([mh Hand] OR palm*:ti,ab OR hand*:ti,ab))).

### Scopus search (629 results)

(INDEXTERMS (“Botulinum Toxins, Type A”) OR INDEXTERMS (Iontophoresis) OR INDEXTERMS (Antiperspirants) OR TITLE-ABS (“botulinum toxin*”) OR TITLE-ABS (“botulinum A toxin*”) OR TITLE-ABS (“botulinum neurotoxin*”) OR TITLE-ABS (Botox) OR TITLE-ABS (Oculinum) OR TITLE-ABS (OnabotulinumtoxinA) OR TITLE-ABS (“Onabotulinumtoxin A”) OR TITLE-ABS (Meditoxin) OR TITLE-ABS (Vistabel) OR TITLE-ABS (Neuronox) OR TITLE-ABS (iontophores*) OR TITLE-ABS (antiperspirant*) OR TITLE-ABS (“aluminum chloride”) OR TITLE-ABS (“topical aluminum chloride”)) AND (CHEM (term) OR TITLE-ABS (“palmar hyperhidros*”) OR TITLE-ABS (“hyperhidrosis palmar*”) OR TITLE-ABS (“sweaty palm*”) OR ((INDEXTERMS (Hyperhidrosis) OR INDEXTERMS (Sweating) OR TITLE-ABS (hyperhidros*) OR TITLE-ABS (sweat*)) AND (INDEXTERMS (Hand) OR TITLE-ABS (palm*) OR TITLE-ABS (hand*)))).
